# News and Miscellany

**Published:** 1901-09

**Authors:** 


### News and Miscellany.

**Rio Chemical Co.** have removed their office from St. Louis to New York City, No. 56 Thomas Street.

**Wanted.**—A physician's residence and location. Price about $800. Address Box 56, Coupland, Texas.

**Saw you** running for a late car last night. Did you catch it?
"Yes; when I got home," he said with a sigh.

**Have you seen** "Ten Nights in a Barroom?" she said.
"No; but I've seen ten barrooms in a night."

**Dr. J. W. McCarver,** graduate of the Medical Department, University of Texas, has been appointed House Surgeon of St. Mary's Hospital, Galveston.

**The State Board** of Medical Examiners for Texas, will meet in Austin October 1, next, for business. For rules, blanks, etc., address Dr. M. M. Smith, Sec., Austin, Texas.

**The boy's** are rattling in renewals for 1901–2, and they nearly all write: "Can't do without the 'Red Back.'" I shall be able to sport a new overcoat by Christmas, I hope.

**Dr. B. F. Church,** formerly of Austin, now President of the Academy of Medicine, San Angeles, Cal., is taking a course in eye, ear, nose and throat at the New York Polyclinic and other p. g. schools.

**I have sixty copies** left of the first edition of my book, "Recollections of a Rebel Surgeon," and as a new edition will be issued from Chicago this month I will sell them at 50 cents each, and ten cents for postage. Speak quick.

F. E. Daniel.

**For Sale.**—$1500 will buy property and practice; extra good location. Have been here 11 years, want to retire, no competition. Two churches, four stores, two gins and mills, good school with 3 teachers. Address M. D., care of this journal.

**Wanted.**—A good location to practice medicine in town or country. Would like to form copartnership with some physician wishing to retire from active practice, or take charge of practice entirely. Twelve years experience. Best references. Address D. M., care TEXAS MEDICAL JOURNAL, Austin, Texas.

---

**The Oklahoma Medical News** is the name of a new medical monthly just launched at Oklahoma City, Ok. It seems to be O. K. I have received Vol. 1, No. 2. Dr. J. R. Phelan is Editor, C. A. Phelan is Business Manager. The *Red Back* extends and wishes the *News* success. A fellow Phelan, you greeting know.

---

**Those of the** "Red Back's" readers who are not acquainted with the merits of Daniel's Concentrated Tincture of Passiflora as a nervous and arterial sedative, especially useful in cerebral hyperaemia and consequent insomnia, are advised to get a bottle and try it. Address Dr. J. B. Daniel, Atlanta, Ga. Chiles & Co. of Austin keep it in stock.

---

**Our friend** and long time patron, Dr. J. A. McGee, of Drs. McGee & Sloan, Rice, Texas, lost his office by fire February last. He laments the loss of his file of Journals, including the *Red Back* from Vol. 1, No. 1. In renewing his subscription for the seventeenth year Dr. McGee writes: "I prized my Journals very highly,—none more than the "Red Back."

---

**The Right Stamp.** We have received from the great Texas State Fair Association, Dallas, and hereby acknowledge with thanks, a date stamp, which we find up to date and very useful in business. It saves labor, and that is a big thing these dog-days. The great Texas State Fair will be held at Dallas Sept. 28.Oct. 13, inclusive. Sidney Smith, Sec. and Gen'l Mgr.

---

**The "Red Back"** learns with much regret that Dr. J. H. Reuss of Cuero, the member from the Eleventh Congressional District on the State Board of Medical Examiners, sustained a vertical fracture of the patella recently, which will confine him some weeks. It is hoped he will be able to attend the meeting of the Board at Austin October 1, and he will do so, even if on crutches. He's a hard man to down!

**New Orleans Polyclinic.**—Physicians will find the Polyclinic an excellent means for posting themselves upon modern progress in all branches of medicine and surgery. The specialties are fully taught, particulary laboratory work. Fifteenth annual session opens November 14, 1901. For futher information address Dr. Isadore, Dyer, Secretary, New Orleans Polyclinic, New Orleans.

---

**A Good Thing.**—Some advertisers know a good thing when they see it. A leading St. Louis firm of manufacturing pharmacists who have one of the "best things out" which they have advertised in the "Red Back" sixteen consecutive years—paying advanced rates four times, in renewing for seventeenth round, write: "We intend to advertise in your journal as long as the journal or ourselves last." (Name on request.)

---

**The Old Confeds.**—The next reunion of the Association of U. C. V.'s and of the Army and Navy medical officers, C. S., will be held in Dallas next June. Bully! I hope to rake up money enough (from delinquent subscribers to the "Red Back") to go, and to get me a long tailed coat to wear at the banquet. Dr. H. A. Moseley of Dallas is chairman of the committee on arrangements. If he doesn't arrange for a banquet I'll have him bounced and put another fellow in who will.

